# Poly(phenylene ether) Based Amphiphilic Block Copolymers

**DOI:** 10.3390/polym9090433

**Published:** 2017-09-08

**Authors:** Edward N. Peters

**Affiliations:** SABIC, Selkirk, NY 12158, USA; ed.peters@sabic.com; Tel.: +1-518-475-5458

**Keywords:** polyphenylene ether, compatibilizer, interface, polybutylene terephthalate, polystyrene, hydrophobic, polyurethane, polyesters

## Abstract

Polyphenylene ether (PPE) telechelic macromonomers are unique hydrophobic polyols which have been used to prepare amphiphilic block copolymers. Various polymer compositions have been synthesized with hydrophilic blocks. Their macromolecular nature affords a range of structures including random, alternating, and di- and triblock copolymers. New macromolecular architectures can offer tailored property profiles for optimum performance. Besides reducing moisture uptake and making the polymer surface more hydrophobic, the PPE hydrophobic segment has good compatibility with polystyrene (polystyrene-philic). In general, the PPE contributes to the toughness, strength, and thermal performance. Hydrophilic segments go beyond their affinity for water. Improvements in the interfacial adhesion between polymers and polar substrates via hydrogen bonding and good compatibility with polyesters (polyester-philic) have been exhibited. The heterogeneity of domains in these PPE based block copolymer offers important contributions to diverse applications.

## 1. Introduction

Poly(2,6-dimethyl-1,4-phenylene ether) (PPE) is an engineering thermoplastic. Engineering thermoplastics comprise a special performance segment of synthetic plastic materials that offer enhanced properties. When properly formulated, they may be shaped into mechanically functional, semi-precision parts or structural components. Mechanically functional implies that the parts may be subjected to mechanical stress, impact, flexure, vibration, sliding friction, temperature extremes, hostile environments, etc., and continue to function.

**Figure 1:**
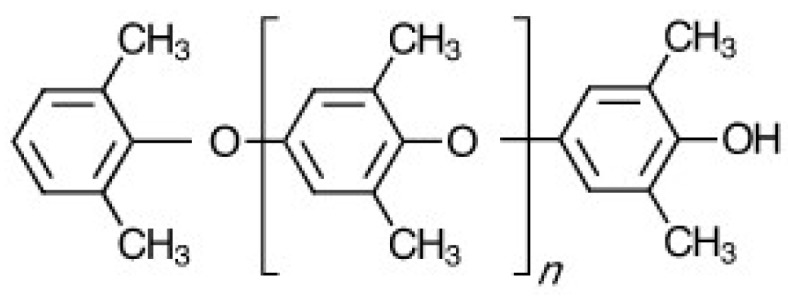
Depiction of poly(2,6-dimethyl-1,4-phenylene ether) (PPE) chemical structure.

The structure of PPE is depicted in Figure 1. The principal repeat unit is the 2,6-dimethyl-1,4-phenylene unit. At opposite ends of the polymer chain is a 2,6-dimethylphenoxy tail-group and a 3,5-dimethyl-4-hydroxyphenyl head-group. It has a highly aromatic structure with a stiff backbone, no hydrolysable bonds and no polarizable groups. These structural features result in a polymer with outstanding hydrolytic stability, very low moisture absorption, high glass transition temperature (Tg), low density, and very low dielectric properties [1,2,3,4,5]. PPE is used primarily in blends and alloys to enhance the thermal performance, increase toughness and lower moisture absorption of other thermoplastics.

**Figure 2:**
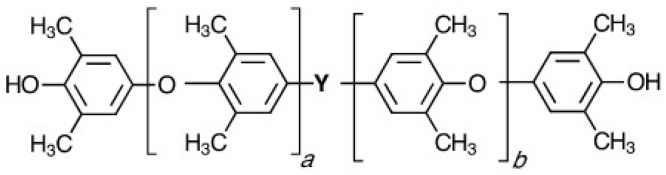
Depiction of PPE telechelic macromonomers (PPE-M) chemical structure.

The very high molecular weight, high melt and solution viscosities, and low number of functional groups per molecule limits PPEs use as a reactive building block. However, unique PPE telechelic macromonomers (PPE-M) have been developed for facile incorporation of PPE features into various copolymers and thermosetting resins [6,7,8,9]. PPE-M has the features of PPE, plus very low molecular weight, which increases its solubility in solvents and monomers and a functionality around two. PPE telechelic macromers have been heralded as a breakthrough in the search for materials that broadly enhanced performance of dielectric materials [6,7,8,9,10,11]. The structure of PPE-M is depicted in Figure 2. Hydroxyl groups at each end of the oligomer facilitate use in thermoset resins and in the synthesis of linear high molecular weight copolymers.

Features of PPE with PPE-M are summarized in Table 1. PPE-M has a significantly lower number average molecular weight (Mn), which translates to greater solubility in solvents. This high solubility, functionality around two, and low viscosity facilitates the use of PPE-M in preparative polymer chemistry. The hydroxyl equivalent weight (HEW) for PPE-M is much lower than that of PPE. The telechelic nature of PPE-M makes it useful as a cross-linker, chain extender, and precursor for block and graft copolymers. The hydrophobicity of PPE-M in thermoplastic and thermoset resins is well documented. In addition, from its engineering thermoplastic heritage and judicious synthesis, PPE-M can increase the thermal properties and toughness, and lower the dielectric properties in thermoplastic and thermoset resins.

In addition to hydrophobicity, the PPE-M domains have other philicities, which contribute to unique property combinations in block copolymers. Amphiphilic block copolymers (ABC) have been prepared with hydrophilic segments such as polyurethanes, polyhydroxyethers (phenoxy resins), and polyether carbonates [12,13,14,15,16,17].

**Figure 3:**
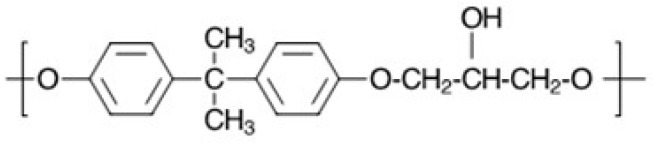
Depiction of polyhydroxy ether (PHE) chemical structure.

Polyhydroxy ethers (PHE) or phenoxy resins are thermoplastics that were an outgrowth of epoxy resin technology [18]. The structure is depicted in Figure 3. Structural features include ether linkages, which provide flexibility, and pendant hydroxyl groups to promote wetting and hydrogen bonding to polar substrates and fillers. The first half of this review focuses on the use of PPE-M to make PPE-PHE amphiphilic block copolymers to enhance their performance and expand their utility.

The second half of this paper reviews the use of PPE-M in thermoplastic polyurethanes (TPUs). TPUs are versatile polymers with many useful properties. However, TPUs can have limited service temperature, high moisture absorption, and poor resistance to burning. Clearly, the engineering features of PPE-M and TPUs would be complementary in a block copolymer and present an opportunity to enhance the performance of TPUs.

## 2. Materials and Methods 

### 2.1. Preparation of Polyhydroxy Ethers

To prepare stable, linear, high molecular weight PPE-PHE ABCs, some important synthetic techniques need to be followed. These critical steps are summarized in a general procedure below. 

The PPE telechelic macromonomer (PPE-M) (Mn ~ 1670) is available from SABIC LLC (Bergen op Zoom, The Netherlands) under NORYL™ SA90 designation. Experimental PPE-M with Mn of 2750 and 1150 were supplied by SABIC LLC (Bergen op Zoom, The Netherlands). The diglycidyl ethers of bisphenol A (DGEBPA) with various epoxy equivalents were obtained from Dow Chemical (Midland, MI, USA) and Momentive Specialty Chemicals (Columbus, OH, USA). The HEW, epoxy equivalent weight (EEW) and the functionality of PPE-M and DGEBPA, respectively, were determined by nuclear magnetic resonance (NMR).

Alternating block copolymers were prepared by the reaction of PPE-M with DGEBPA. The molecular weights of the PHE segments were calculated by the EEW × functionality. The molecular weights of the PPE segments were calculated by the HEW × functionality. The number average molecular weight of the PPE block and the PHE was varied from 1150 to 2750 and 400 to 5000, respectively. The PPE content in the block copolymers was varied from 24 to 80 wt %.

**Figure 4:**
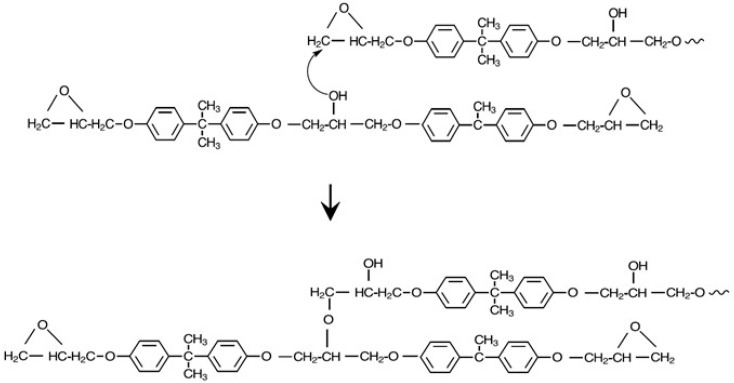
Branching reaction in diglycidyl ethers of bisphenol A (DGEBPA).

Key consideration for preparing linear, high molecular weight block copolymers include 1:1 stoichiometry of phenolic to epoxy groups and reactants with a functionality of 2. For good thermal stability, the final polymer should not have any unreacted epoxy end groups. Another important consideration is avoiding the side reactions inherent in epoxy resin chemistry where secondary alcohol groups react with epoxy groups, leading to branching [19,20]. For example, up to 11.9% branching has been reported in phenoxy resins prepared from the reaction of BPA with DGEBPA [21]. The branching reaction is shown in Figure 4. 

**Figure 5:**
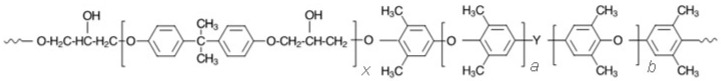
The general structure of PPE-PHE block copolymer.

The reaction of the PPE-M phenolic groups with epoxy is significantly faster than the reaction of alcohol groups with epoxy [11]. Therefore, the synthesis involved programed addition of the DGEBPA to a solution of PPE-M so that there was always an excess of phenolic groups. The PPE-M was dissolved in cyclohexanone at 150 °C. Using 0.1 mole % *N*,*N*′-dimethylamino pyridine as a catalyst, the epoxy was added over 5–6 h. After cooling, the block copolymer was isolated by precipitation in methanol and dried. This procedure resulted in linear polymers with <0.1% branching. The structure appears in Figure 5. ^1^H-NMR 400 MHz monitored the disappearance of methine hydrogen on the oxirane and the appearance methine hydrogen of secondary alcohol using. NMR confirmed the absence of any unreacted epoxy. 

### 2.2. Blends and Composites

PBT (poly(butylene terephthalate)) was obtained as VALOX™ 315 from SABIC LLC. PPE, having an intrinsic viscosity of about 0.40 dL/g (deciliter per gram) as measured in chloroform at 25 °C was obtained as PPO™ 640 from SABIC LLC. SEBS, polystyrene-poly(ethylene-butylene)-polystyrene triblock copolymer having a polystyrene content of about 30% was obtained as KRATON™ G1650M from Kraton Performance Polymers Inc (Houston, TX, USA). PHE resin was obtained as PKHH™ from Gabriel Performance Products (Akron, OH, USA). Formulations are summarized in Table 2.

PS (atactic polystyrene), was obtained as STYRON™ 685DL from Americas Styrenics LLC (Torrance, CA, USA). Chopped glass fiber (GF) having a diameter of about 10 micrometers, a length of about 3.2 mm, and a γ-amino-propyl-silane surface treatment was obtained as E-Glass Chopped Strand from Nippon Electric Glass Company (Otsu, Japan). Formulations are summarized in Table 3.

Blends were compounded on a Coperion ZSK 18 twin-screw laboratory extruder (18 mm screw outer diameter) operating at a screw rotation rate of 300 revolution per minute and zone temperatures of 180, 230, 260, 270, 270, 270, and 270 °C from feed throat to die. Parts for physical property determination were obtained by injection molded using a Demag Plastic Group Model 40–80 injection molding machine (Strongsville, OH, USA).

### 2.3. Preparation of Thermoplastic Polyurethanes

Relative to typical polyurethane building blocks, the PPE-M has phenolic end groups and is a solid with a high Tg and softening point as indicated in Table 1. Some challenges for the use of PPE-M to prepare PPE-based TPUs were how to handle the solid PPE-M in typical urethane synthetic procedures and understanding the reactivity of the PPE phenolic end group with isocyanates. Below a general procedure summarizes the process and chemistry details for the facile synthesis of PPE-based TPUs.

Ethylene oxide-capped oxypropylated polyether diol, EO-PO, (OH = 56.7 mgKOH/g) was Poly-G™ 55–56 supplied by Arch Chemicals, Inc (Norwalk, CT, USA). 4,4′-Diphenylmethane diisocyanate, 4,4′-MDI, (NCO% = 33.6%, eq. wt. 125.06) was Mondur™ M from Covestro AG (Leverkusen, Germany). The chain extender was 1,4-butandiol, BD, (Eq. wt. = 45) from Alfa Aesar (Ward Hill, MA, USA). Dibutyl-tin dilaurate, DBTDL, catalyst was Dabco™ T-12 from Air Products (Allentown, PA, USA).

The solubility/compatibility of PPE-M was determined in a wide variety of polyols and chain extenders. In general, the PPE-M exhibited good solubility and compatibility with polyether polyols. Soluble, homogeneous blends of PPE-M and EO-PO were liquids with Tgs below room temperature. These homogeneous liquid blends provided a convenient route for incorporation of PPE-M into the TPU polymerization process.

**Figure 6:**
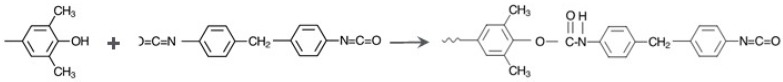
Reaction of PPE-M with isocycanate group.

**Figure 7:**
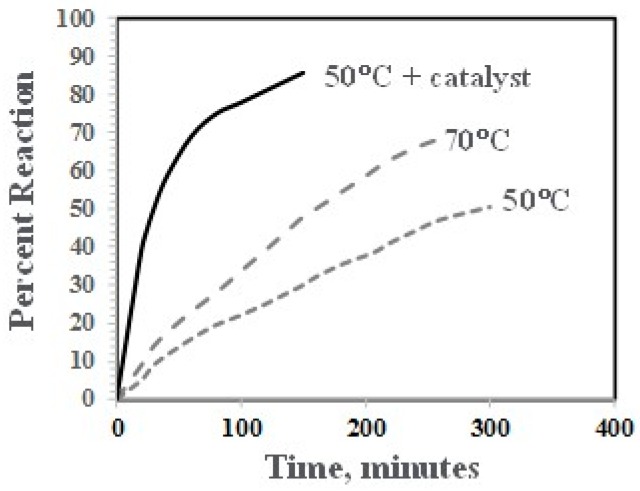
Rate of reaction of PPE-M with MDI.

The reaction of the phenolic groups of PPE-M with isocyanate groups is depicted in Figure 6. The rate of reactions of PPE-M with 4,4′-MDI (0.13 mol/kg and 0.15 mol/kg, respectively) were measured in toluene at 50 °C and 70 °C. The reactions were monitored by measuring the concentration of unreacted isocyanate group via standard di-n-butylamine titration method [22]. The PPE-M readily reacts with the isocyanate groups. The reactions followed second order kinetics up to high degree of isocyanate conversion. The reaction mixtures remained as a single phase liquid during the kinetic measurements. The rate of reaction increased with increased reaction temperature. In addition, the use of DBTDL (0.01%) resulted in a significant increase in the rate. Kinetic data is shown graphically in Figure 7.

The formulations for preparing PPE-M/EO-PO based TPUs appear in Table 4. The object was to determine the performance features of TPUs as the hydrophobic content changed with increasing amounts of PPE-M. The weight ratio of PPE-M to EO-PO was 0/100, 10/90, 20/80, and 30/70 and the content of hydrophobic PPE-M segments was 0, 7.65, 15.29, and 23 weight %. There was no attempt to optimize any of the formulations.

**Figure 8:**
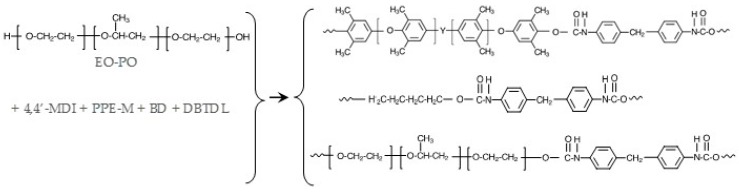
General structure of segments in PPE-M based TPUs.

TPUs were prepared via bulk polymerization, where the PPE-M was pre-dissolved in the polyol. At 100 °C, MDI (warmed to 80 °C) was added via syringe to the mixture of polyols, BD, and DBTDL catalyst (~0.003 phr) and mixed via a Speed Mixer (2200 rpm) for 30–40 s and then transferred to a preheated mold at 120 °C. The TPUs were cured for 2 h at 120 °C and then post-cured for 20 h at 100 °C. FTIR spectra of cured TPUs indicated that polymerizations were completed; there was no or only traces amounts of unreacted isocyanate (-NCO groups) at 2270 cm^−1^. The general synthesis of PPE-M/EO-PO based TPUs are shown in Figure 8.

## 3. Results

### 3.1. Properties of PPE-PHE Block Copolymers

**Figure 9:**
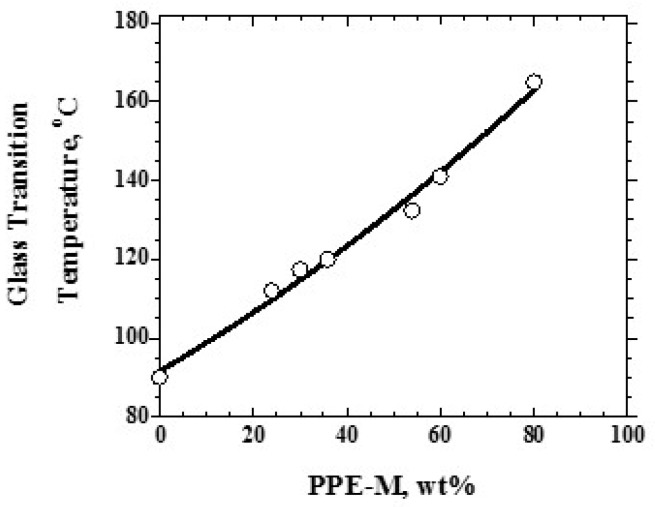
Tg versus PPE-M (Mn 1670) Content.

**Figure 10:**
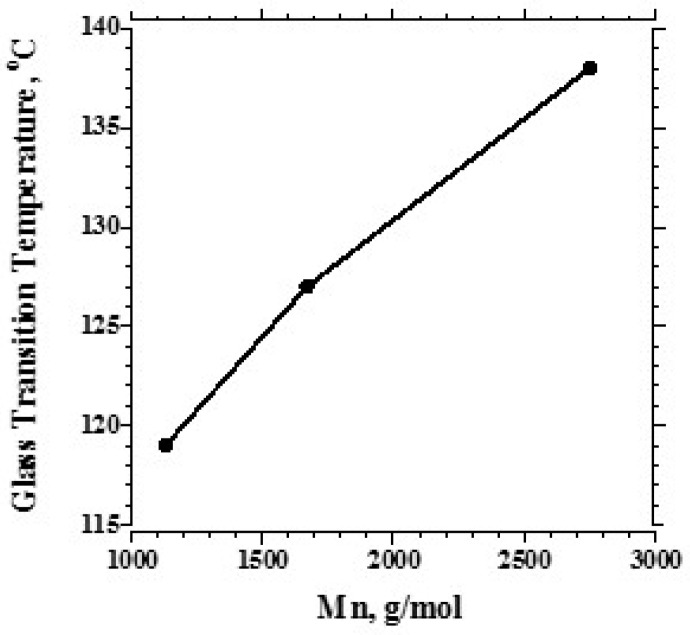
Tg versus Mn of PPE-M segments (48 wt %).

Over the range studied using PPE-M with a Mn of 1670, the PPE-PHE block copolymers exhibited a single Tg which increased with increased levels of PPE-M as shown in Figure 9. PHEs have relatively low Tgs around 90 °C and end use temperatures are restricted to about 60–80 °C [12]. With the higher Tgs (>100 °C), PPE-PHE block copolymers offer opportunities in adhesives and coatings where >80 °C use temperatures are required. The effect of molecular weight on Tg was studied in copolymers containing about 48 wt % PPE-M. The results in Figure 10 show a single Tg which increased with increased molecular weight of PPE-M. Except where noted, most of the evaluations in this review were done with PPE-PHE block copolymers with PPE-M with a Mn of 1670.

**Figure 11:**
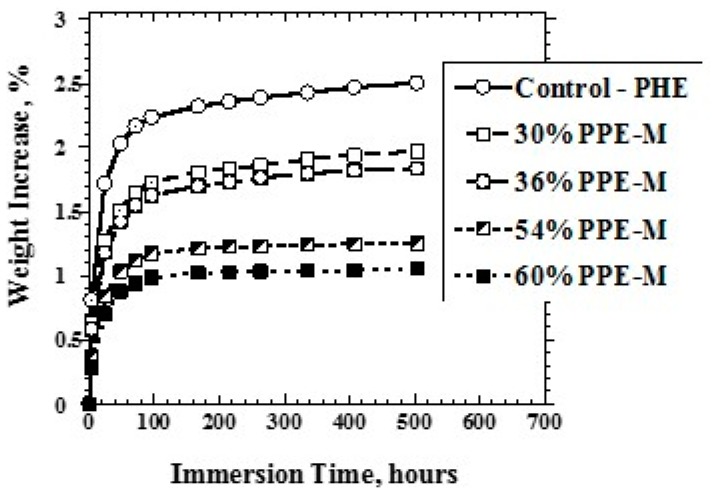
Water uptake versus PPE-M content.

Water absorption in polymers is known to have adverse effects on dimensional stability, Tg, and mechanical properties. The alcohol groups on PHE can hydrogen bond to water. However, the PPE segments do not contain polar groups, which would strongly hydrogen bond to water, and, correspondingly, have very low water absorption. PPE-PHE block copolymers immersed in water at 80 °C exhibited weight increases over time. As shown in Figure 11, there was a significant decrease in water absorption with increasing levels of PPE-M.

**Figure 12:**
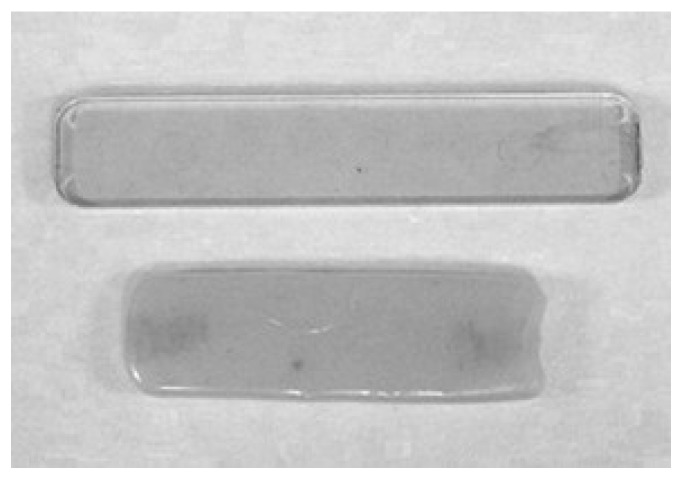
Test parts before and after 90 h immersion in water.

Interestingly, the PHE control sample distorted in less than 90 h of immersion, as shown in Figure 12. This suggests that the absorbed water plasticized the PHE and depressed the Tg below 80 °C. Hence, the part distorted.

**Figure 13:**
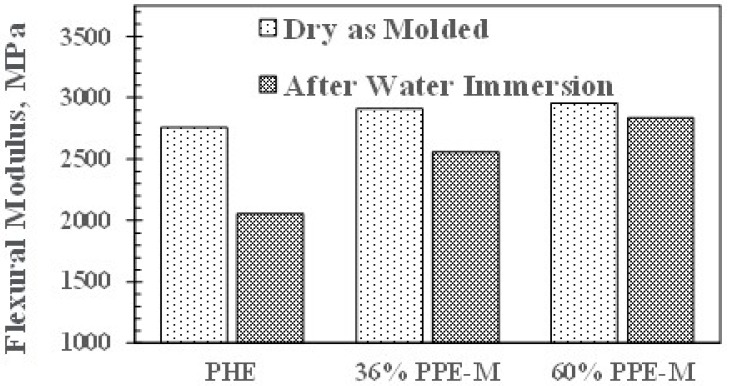
Effect of water uptake on flexural modulus.

In dry samples, the flexural modulus (FM) increased slightly with increasing PPE-M content. However, after 16 h immersion in water at 80 °C, the FMs decreased. This corresponds to moisture uptake which acts as a plasticizer. PHE exhibited the greatest decrease. The PPE-PHE block copolymers exhibited much greater retention of FM as shown in Figure 13.

**Figure 14:**
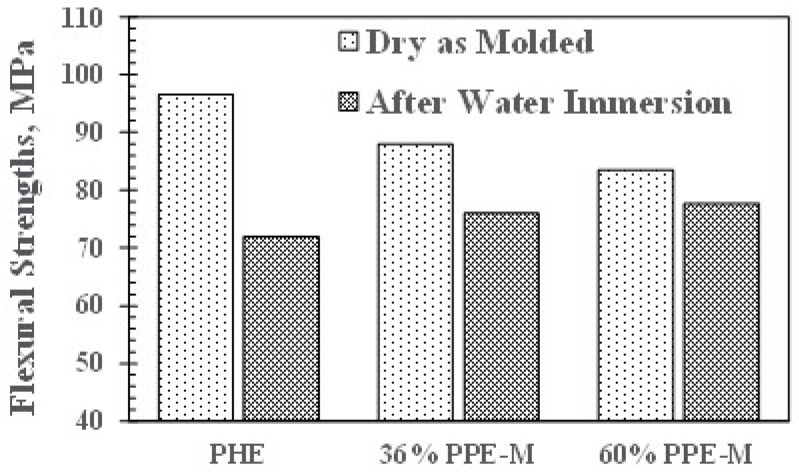
Effect of water uptake on flexural strength.

Flexural strengths (FS) in dry samples exhibited a slight decrease with increasing PPE-M content. The FSs decreased after 16 h immersion in water at 80 °C. PHE exhibited the greatest decrease in FS. The PPE-PHE block copolymers exhibited much greater retention of FS than the PHE as shown in Figure 14. 

### 3.2. Blends, Alloys, and Composites of PPE-PHE Block Copolymers

The PPE-PHE amphiphilic block copolymers represent an interesting class of polymers that have other useful philicities. High molecular weight PPE and PHE polymers will form miscible blends with polystyrene and polyesters, respectively. Hence, the PPE block is polystyrene-philic and the PHE block is polyester-philic. In addition, the PHE contains hydroxyl groups to facilitate hydrogen bonding. The polyphilic nature of these block copolymers can offer utility as interfacial agents in polymer blends and alloys and in composites [23,24].

Most polymer–polymer blends usually form immiscible blends that have unacceptable properties [25]. In addition, their morphology is unstable and can change with processing conditions. Compatibilization technology is used to improve the interfacial adhesion between the two phases, enhance properties, and give a stable morphology [25,26,27]. Block or graft copolymers with the appropriate structure can be used to promote adhesion at the interface [25,26,27,28,29]. Amphiphilic block copolymers can be useful compatibilizers, modifiers, adhesion promoters, and interfacial agents. The PPE-PHE ABC would have an affinity for two different types of environments. The polyester-philic and PPE-philic segments suggest utility as a compatibilizing agent in polyester/PPE blends.

#### 3.2.1. Alloys with Poly(butylene terephthalate)

In this review, the term polymer alloy will refer to an immiscible polymer blend having a modified interface and/or morphology [25,27,28,29]. Alloys of semi-crystalline and amorphous polymer are particularly attractive. The key to unique alloys is to leverage key attributes from both polymers. In general, semi-crystalline polymers have chemical resistance and low melt viscosity. Amorphous polymers can provide dimensional stability, freedom from warpage, and improved impact strength [25]. Alloys of poly(butylene terephthalate) (PBT) and PPE are of particular interest. Features of PBT include chemical resistance and good processability, however, the Tg around 34 °C results in dimensional changes at relatively low temperatures [30,31]. PPE has a Tg above 200 °C, good dimensional stability and properties at elevated temperatures, but is more difficult to process [30,31].

PPE blends with polyesters are immiscible [30,31]. Hence, compatibilization technology is essential for improving the interfacial adhesion between the two phases to give a stable morphology and in achieving broad enhancement of useful properties.

In polymer blends, PHE forms miscible blends with a variety of polar polymers, such as aliphatic and semi-aromatic polyesters [32,33,34,35,36,37]. The secondary alcohol groups on PHE can take part in interactions with polar polymers, which seem to be in the origin of miscibility and compatibility with polar polymers [38,39]. PHE forms miscible blends with PBT [39]. However, PHEs are incompatible with non-polar polymers such as PPE [40]. Thus, the unique PPE-PHE ABC has segments that would facilitate attraction to both phases.

PBT/PPE compositions were summarized in Table 2. All blends contained 40 wt % PBT. The 10 wt % SEBS impact modifier would reside in the PPE phase.

Control compositions included PBT by itself and the use of PHE as a compatibilizer. The ABC made with 36 wt % PPE-M was used in these evaluations and has the designation ABC-36. In general, polyesters contain a transesterification catalyst. Therefore, to prevent PBT-PHE catalyzed exchange reactions, anhydrous sodium phosphate monobasic (0.3 wt %) was added to quenching the catalyst [41].

**Figure 15:**
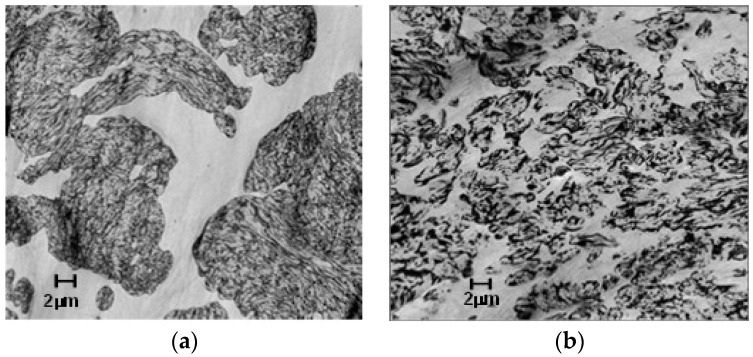
Morphology of poly(butylene terephthalate)-PPE (PBT-PPE) blends: (**a**) uncompatibilized; and (**b**) compatibilized with amphiphilic block copolymers (ABC-36).

Microscopy (STEMs 2000×) shows the effectiveness of the PPE-PHE block copolymers as a compatibilizer between PPE and PBT phases. PBT is the continuous phase (light) and the PPE/SEBS is the dispersed domains (dark). The morphology of PBT/PPE/SEBS control in Figure 15a shows very large PPE/SEBS domains. On the other hand, the morphology of alloy containing ABC-36 in Figure 15b reveals significantly smaller, well-dispersed PPE/SEBS domains. These micrographs suggest that the ABCs are interacting with both PBT and PPE phases, which should translate to enhancement of properties.

**Figure 16:**
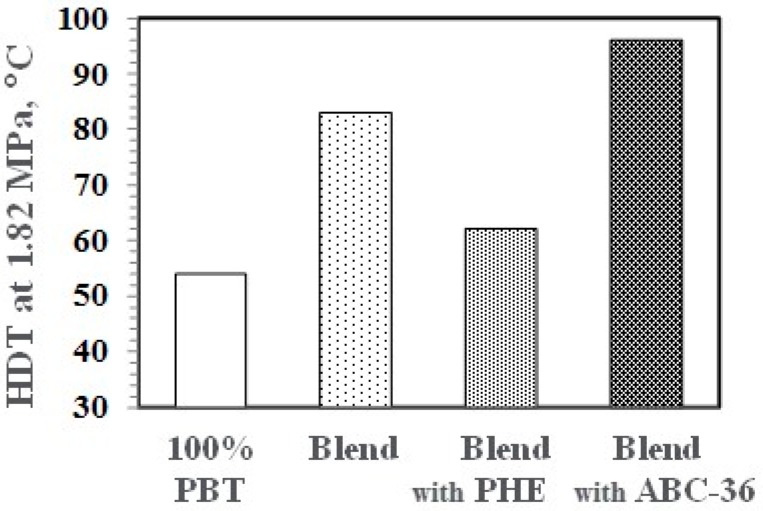
Heat deflection temperature (HDT) of PBT and PBT/PPE Blends.

A hallmark of PPE blends and alloys is the increase in thermal properties. As seen in Figure 16, PBT has a low heat deflection temperature (HDT) at 1.82 MPa. However, the PBT/PPE/SEBS blends with and without ABC-36 exhibited significant increases in the HDT. The HDT of the blend with PHE decreased which could be the result of the lower Tg of PHE.

**Figure 17:**
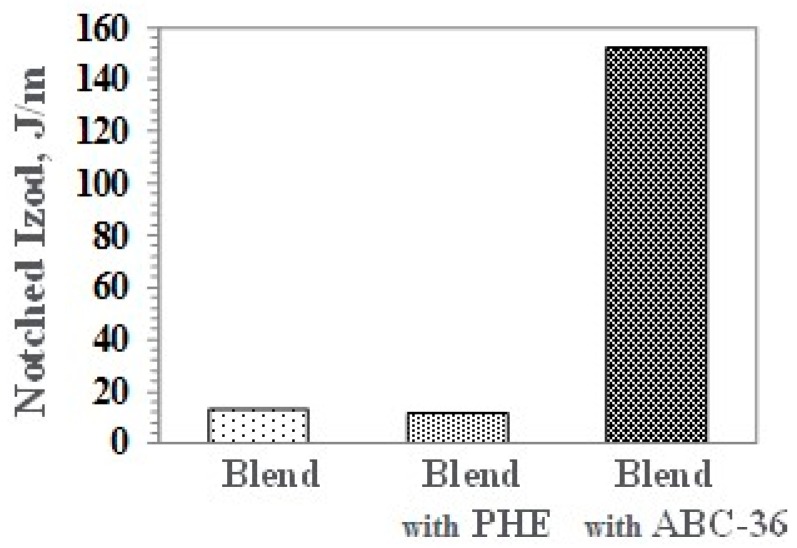
Notched Izod (NI) of PBT/PPE/SEBS Blends.

Notched Izod (NI) impact strength for the blend containing ABC-36 is significantly higher than without any compatibilizer or PHE, as shown in Figure 17.

**Figure 18:**
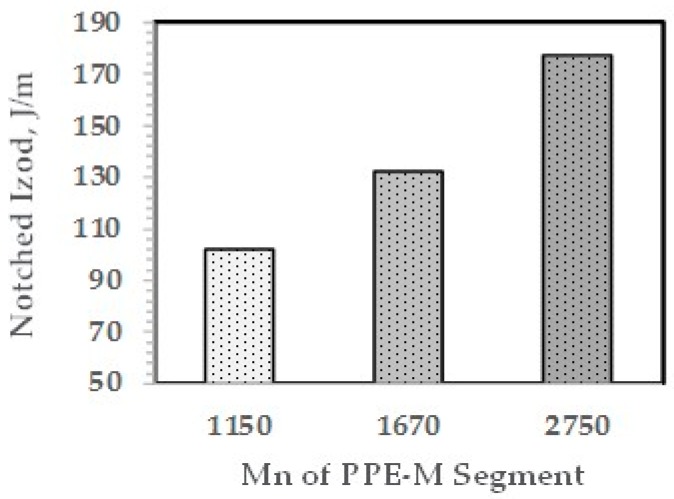
NI versus Mn of PPE-M segments in ABC.

The effect of the molecular weight of the PPE-M segments in the ABC on impact strength appears in Figure 18. The Mn of the PPE-M segments was 1150, 1670, and 2750 and the PPE-M content in the ABC was 48 wt %. All alloys were made with 10 wt % ABC. The impact strength increases with increasing length of the PPE segment. This suggests increased interaction between the PPE-M segments of the ABC and the PPE dispersed phase. 

**Figure 19:**
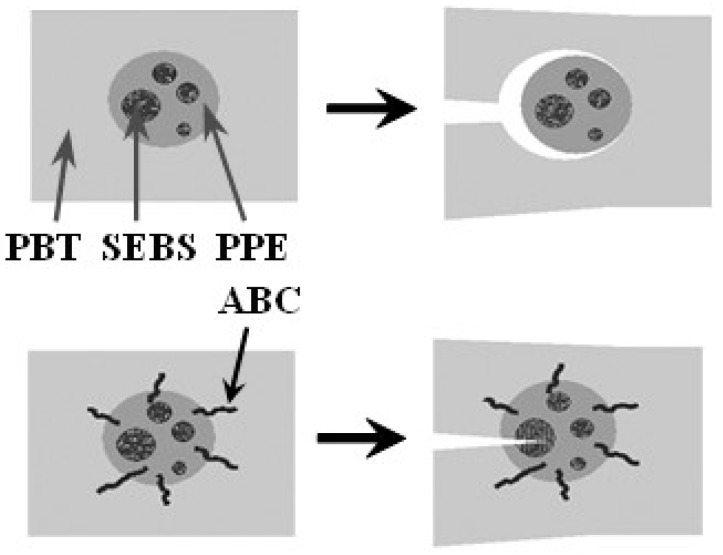
Toughening mechanism.

A possible mechanism of property improvement with the use of ABCs is depicted in Figure 19. Without the PPE-PHE block copolymers, there is essentially little to no adhesion between PBT and PPE phases. Under stress a crack would propagate through the continuous phase and around the PPE/SEBS domain. However, with PPE-PHE block copolymers there is increased interfacial adhesion between the phases, the crack would propagate into the PPE/SEBS domain, and the stresses dissipated.

**Figure 20:**
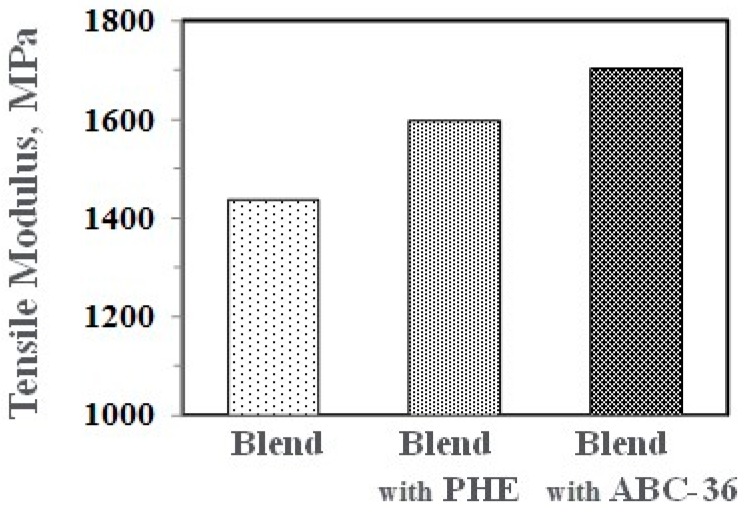
Effect of ABC on tensile modulus.

**Figure 21:**
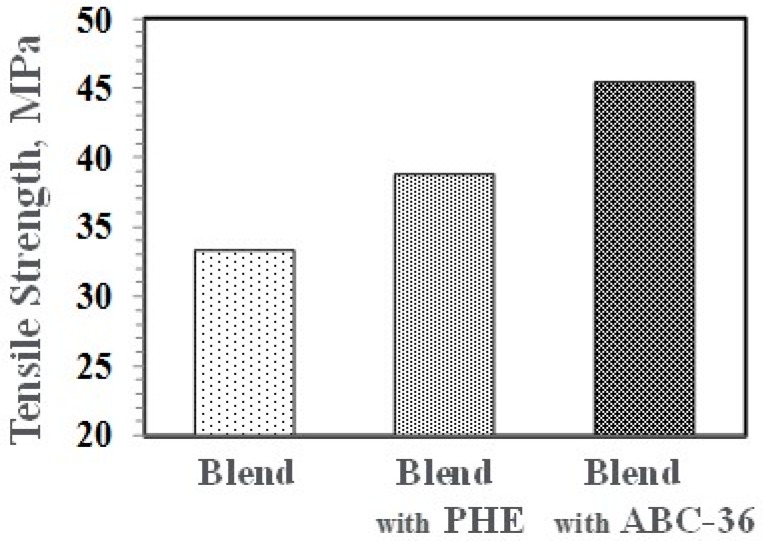
Effect of ABC on tensile strength.

**Figure 22:**
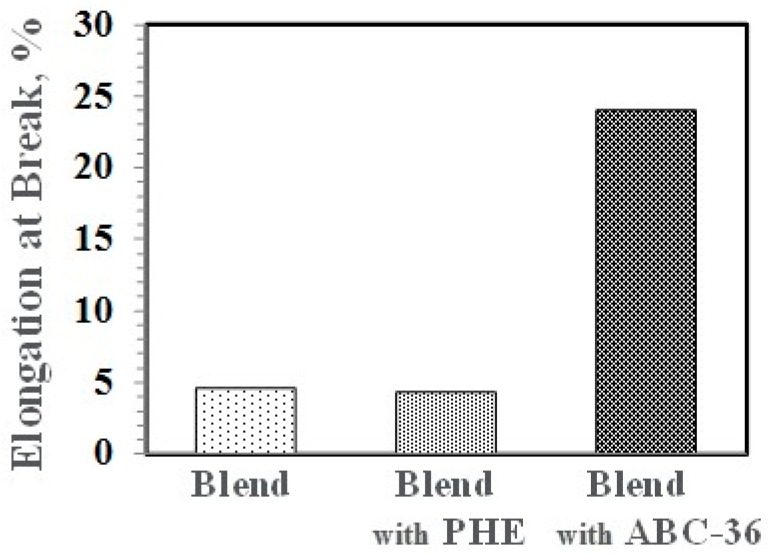
Effect of ABC on elongation at break.

Performance enhancements from the PPE-PHE block copolymers were noted in tensile properties. Tensile modulus and strength increased with the addition of PHE and ABC-36 as shown in Figure 20 and Figure 21, respectively. The increases were greater when ABC-36 was used as a compatibilizer. While the PHE gave properties better than the uncompatibilized PBT/PPE/SEBS blend, the property enhancements were less than those from the ABC-36 composition. This effect of PHE may be due to weak hydrogen bonding with the PPE dispersed phase. Looking at uniaxial stress in Figure 22, the ABC-36 material had significantly higher elongation at break compared to the blend only or with PHE containing blend.

#### 3.2.2. Interface in Composites—Glass Reinforced Polystyrene

The addition of reinforcing fillers and glass fibers to polymers can produce significant improvements in mechanical properties. The reinforcing additives can have geometries such as fibers, flakes, spheres, and particulates. Reinforced polymers would consist of two or more components and two or more phases. Ideally, any applied stresses would be transmitted from the continuous polymer matrix to the reinforcing material. The efficient transfer of stress from the matrix to the dispersed reinforcing material can increase the system ability to absorb energy and translate to increased mechanical properties. Clearly, interphase attraction between reinforcing material and polymer matrix is of paramount importance. Indeed, the mechanical properties of fiber-reinforced composites largely depend on the interphase between the reinforcement and the matrix. 

Some chemical compounds that are used to increase the polymer matrix attraction to a filler surface are called coupling agents. The most common coupling agents are organo-silanes [42]. Hydrogen bonding is an important mechanism to enhance the polymer–filler interface [43].

Optimizing performance via good adhesion between the matrix and fiber is a challenge in glass fiber reinforced polystyrene. In general, glass fiber, GF, contains a coupling agent on the surface to facilitate fiber-matrix attraction. One of the most common agents is γ-amino-propyl-silane. However, in non-polar polymers such as polystyrene there are no opportunities for hydrogen bonding or other interactions with GF.

The amphiphilic PPE-PHE block copolymers were evaluated as additives for increasing the interphase attraction between fiber and PS. The PPE segments polystyrene-philic and the PHE segments contain hydroxyl groups to facilitate hydrogen bonding.

**Figure 23:**
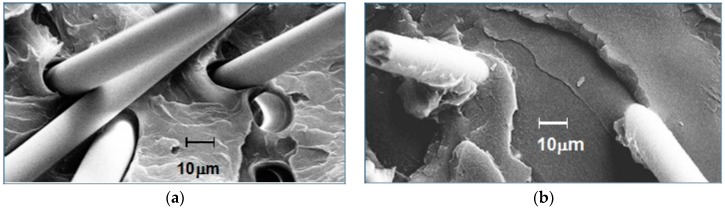
Scanning electron microscope (SEM) images of GF-PS interfaces: (**a**) no interfacial agent; and (**b**) ABC-36 as interfacial agent.

The resin–fiber interface on fractured surfaces of polystyrene containing 20% glass fiber was examined by microscopy. The effects of 0% and 5% ABC-36 are compared by scanning electron microscope (SEM) in Figure 23. In Figure 23a, there is no indication of any resin interaction with the GF. There is no PS adhering to GF and there is separation of the GF from the PS at the base of the GF. On the other hand, the SEM in Figure 23b for polystyrene containing 5 wt % ABC-36 shows resin adhering to the GF and at the base of the fiber there is good resin–fiber adhesion. These SEM results suggest that the ABC increased the interfacial adhesion between the GF and the PS. The PHE segments can hydrogen bond to the GF surface and the PPE segments are soluble in the PS.

**Figure 24:**
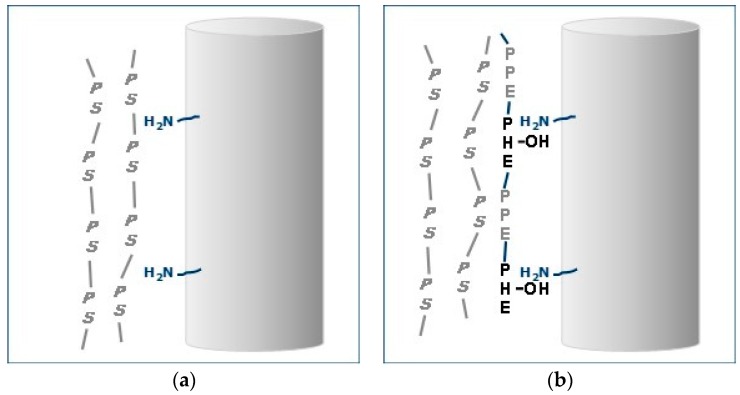
Depiction of PS-GF Interfaces: (**a**) no interaction and no adhesion; and (**b**) ABC at interface and good adhesion.

This effect of ABC on interfacial adhesion is depicted in Figure 24. In the PS–GF blend there is no interaction between the non-polar PS and the polar moieties on the GF surface (Figure 24a). On the other hand, with ABC, the PHE segment would have greater affinity of the polar moieties on the GF and could hydrogen bond with the amino-silanes on the surface (Figure 24b). In addition, the PPE segments would be soluble in the PS. This increase adhesion at the interface translates to increase in properties. 

**Figure 25:**
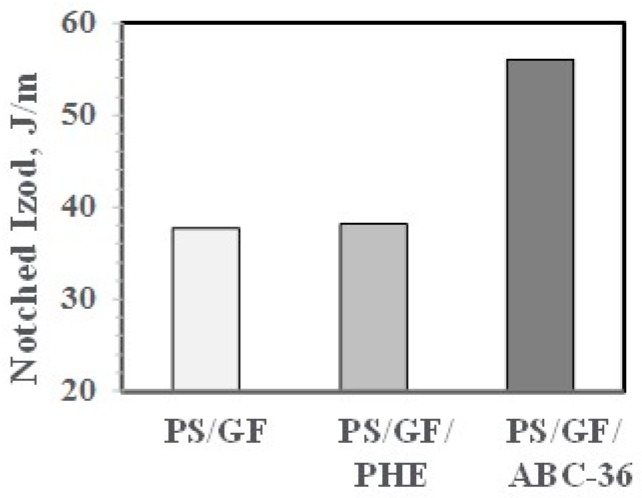
Effect of ABC on NI.

In control blends, PS/GF and PS/GF with 5 wt % PHE, the NIs were low. However, with 5 wt % ABC-36, the NIs were substantially higher, as shown in Figure 25. This suggests that the good resin–fiber adhesion from utilizing the ABC can better transfer any applied stresses from the PS phase to the GF.

**Figure 26:**
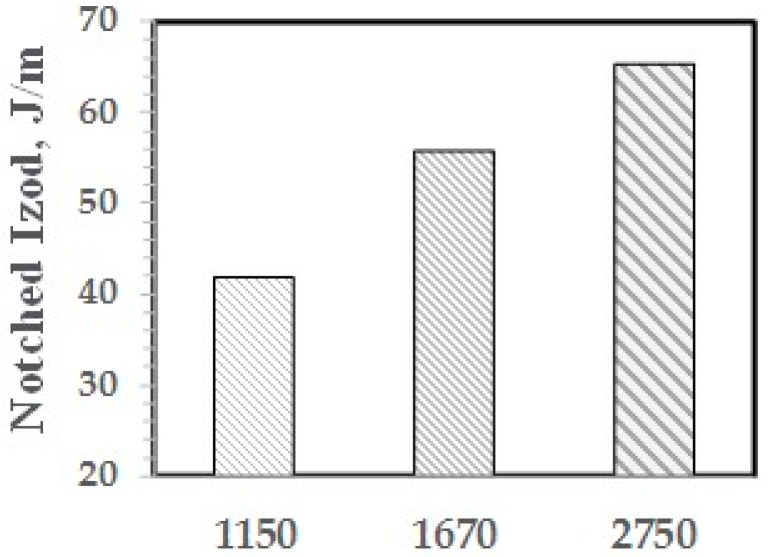
NI versus Mn of PPE-M segments.

The effect of the molecular weight of the PPE-M segments in the ABC on impact strength appears in Figure 26. The ABC contained 48 wt % PPE-M segments and the Mn ranged from 1150 to 2750. All blends contained 5 wt % ABC and 20 wt % GF. The impact strength increased with increasing length of the PPE segment. This suggests that the higher molecular weight segments gives increased adhesion between ABC and PS. The amount of PHE segment and the secondary alcohol levels is essentially unchanged.

**Figure 27:**
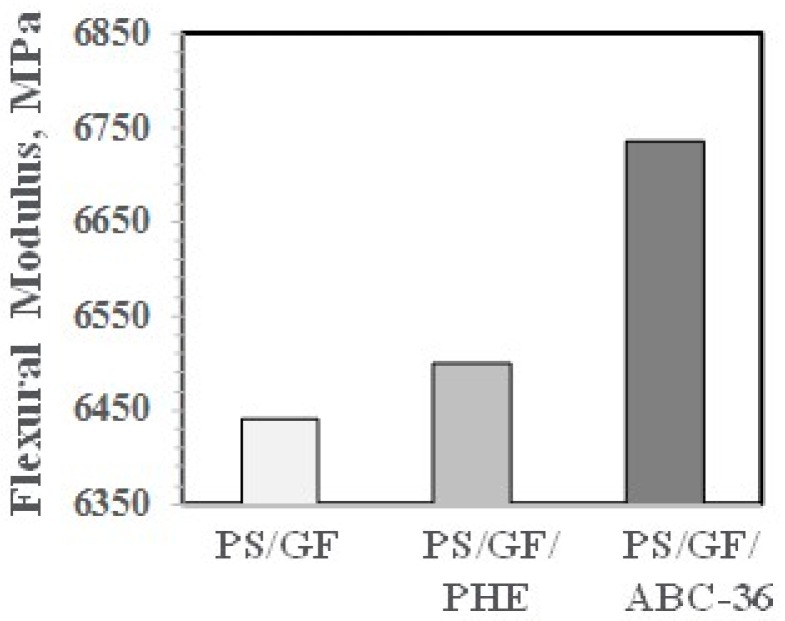
Flexural modulus versus ABC.

**Figure 28:**
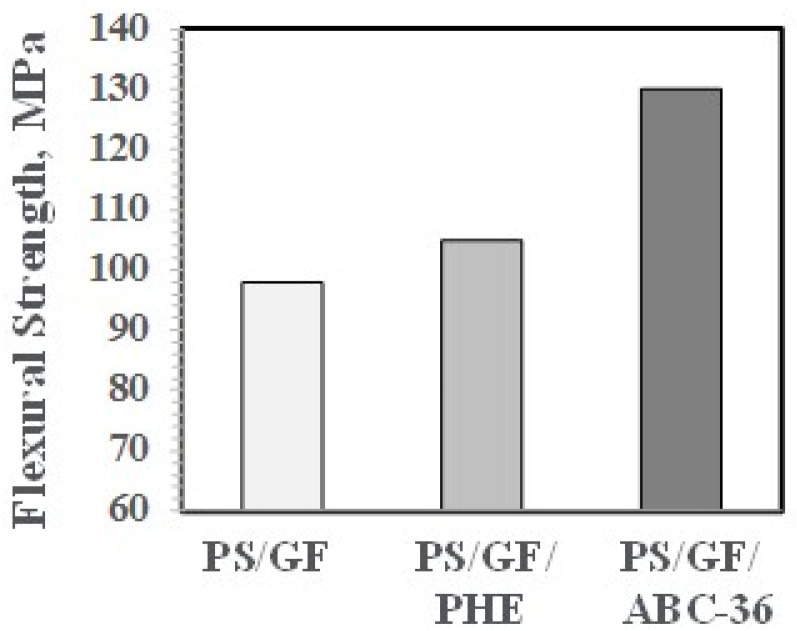
Flexural strength versus ABC.

The effects on PHE and ABCs on the flexural properties appear in Figure 27 and Figure 28. The ABC blends are more effective than PHE in enhancing the FM and FS above the values for PS/GF.

**Figure 29:**
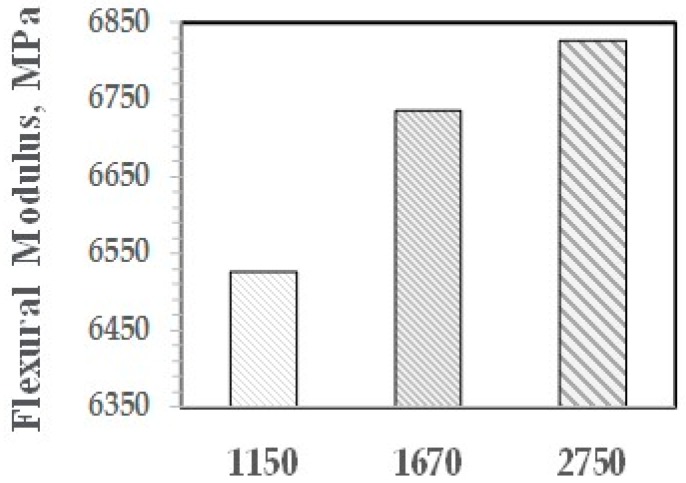
Flexural modulus versus Mn of PPE-M Segments.

**Figure 30:**
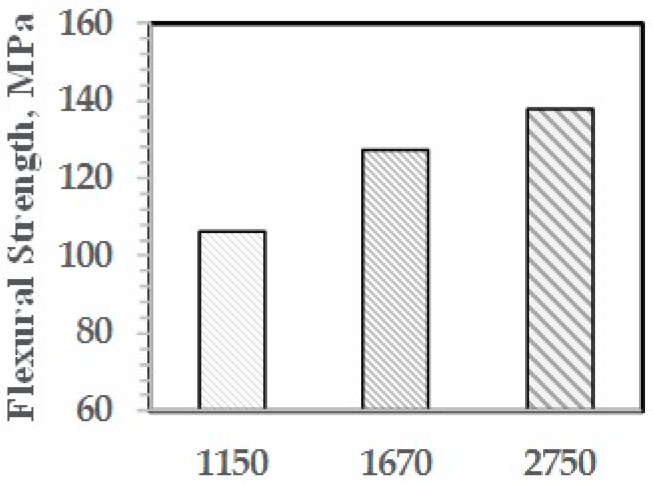
Flexural strength versus Mn of PPE-M segments.

As shown with NI, the higher is the molecular weight of the PPE-M segments, the greater is the increase in properties. The effect of PPE Mn on FM and FS are shown in Figure 29 and Figure 30, respectively.

**Figure 31:**
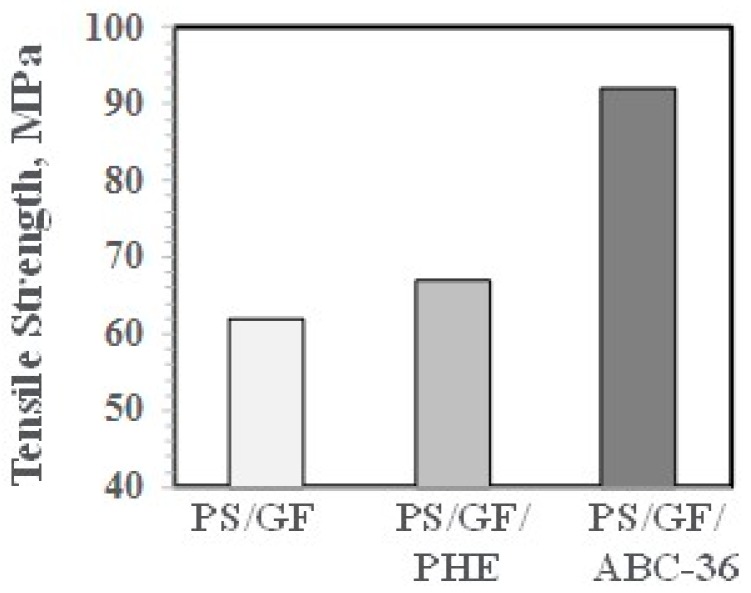
Tensile strength versus ABC.

TS are higher with ABC-36, as shown in Figure 31. These enhanced physical properties suggest that the ABCs are effective in increasing the interfacial adhesion between the PS and GF.

**Figure 32:**
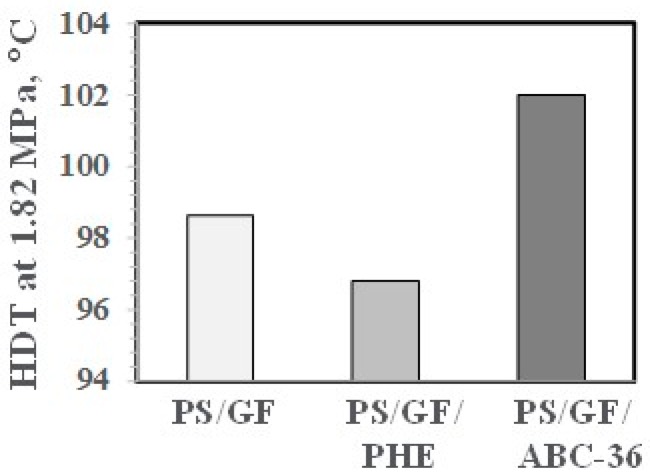
HDT of PS/GF Blends.

Values for HDT results appear in Figure 32. The HDT was much higher with 5 wt % ABC-36. This may be due in part to the Tg of 121 °C for ABC-36 compared to a Tg of 100 °C for PS. The higher HDT is in line with the higher modulus of the ABC based blends and the report that there is a direct correlation of the HDT of amorphous polymers with their modulus behavior of the sample [44,45]. The HDT for 5 wt % PHE was lower than for PS/GF. This may be related in part to the lower Tg of 91 °C for the PHE. 

### 3.3. Thermoplastic Polyurethanes

TPUs are versatile polymers with many useful properties. These inherently flexible polymers are renowned for high tensile elongation, resistance to oil, grease, and certain chemicals and solvents. These characteristics make TPU extremely popular across a range of markets and applications. However, features also include limited service temperature, high moisture absorption, and poor resistance to burning. Clearly, the features of PPE-M and TPUs are complementary and suggest an opportunity to enhance performance of TPUs with PPE-TPU amphiphilic block copolymers. Thus, PPE-M was used as a hydrophobic polyol to prepare TPU amphiphilic block copolymers where the content of the PPE segments were 0, 7.65, 15.3, and 23 wt %. 

**Figure 33:**
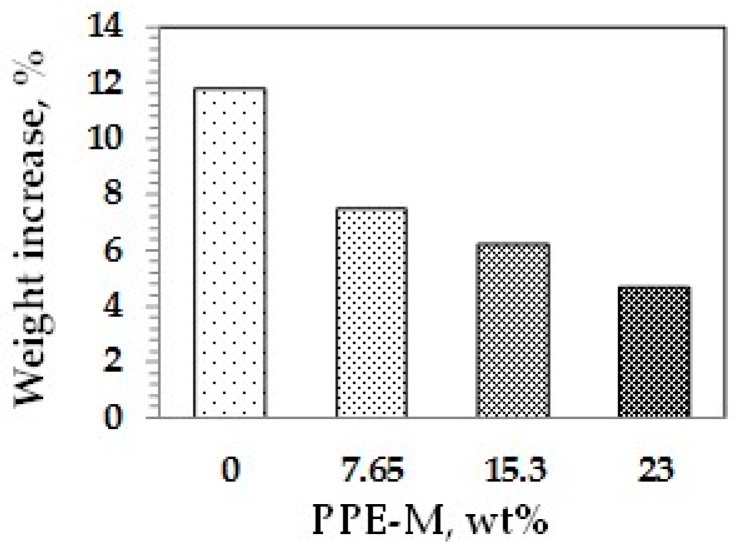
Water uptake after three-day immersion in water.

**Figure 34:**
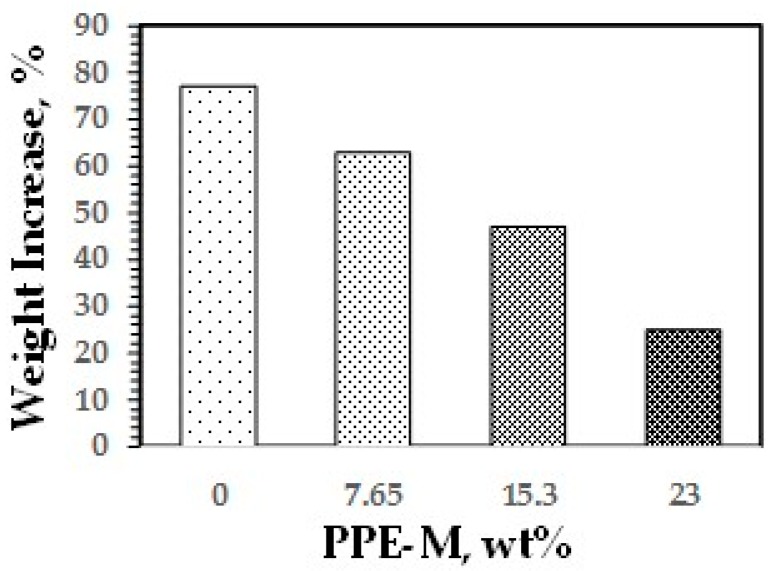
Moisture uptake at 50 °C and 100% RH.

Water absorption in polymers is known to have adverse effects on dimensional stability, Tg, mechanical properties, and dielectric properties. Urethanes can form hydrogen bonds to substrates through the polar urethane group [46,47]. While the urethane moiety can hydrogen bond to substrates to increase adhesion, it can also strongly hydrogen bond with water. However, PPE does not contain polar groups, which would strongly hydrogen bond to water, and, correspondingly, has very low water absorption. The effect of PPE-M on moisture uptake in TPUs was examined by the weight change after exposure to 100% relative humidity (RH) at 50 °C for seven days and immersion in water at ambient temperature for three days. As shown in Figure 33 and Figure 34, the TPU without any PPE-M absorbs high levels of water. However, with increasing amounts of PPE-M, there was a significant decrease in water absorption.

**Figure 35:**
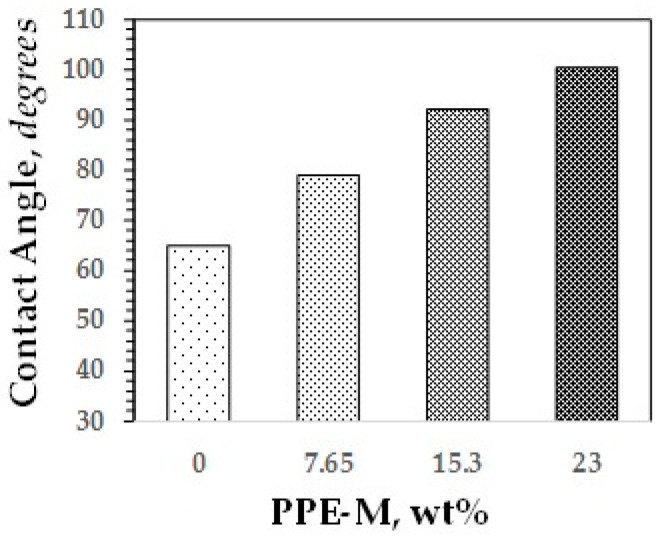
Contact angles of versus PPE-M content.

Water contact angles are considered as a measure of hydrophobicity of a surface. In general, if the water contact angle is larger than 90°, the surface is considered hydrophobic and, if the water contact angle is smaller than 90°, the surface is considered hydrophilic [48,49]. As shown in Figure 35, the hydrophobicity of the TPUs increased with increasing levels of PPE-M. Indeed, around 15.3 wt % PPE-M the contact angle is 92°.

Other hydrophobic diols and technologies have been used to prepare hydrophobic TPUs. These include the use of polybutadiene diols [50,51], α,ω-dihydroxypoly(dimethylsiloxane) [52] and fluorinated macrodiols [53]. In addition, plasma treatment of the surface of TPUs has been used to increase the hydrophobicity [54]. However, these hydrophobic diols lack the engineering features of PPE-M. Butadiene segments are prone to oxidation and, in general, siloxanes have poor tear, abrasion and tensile strength.

**Figure 36:**
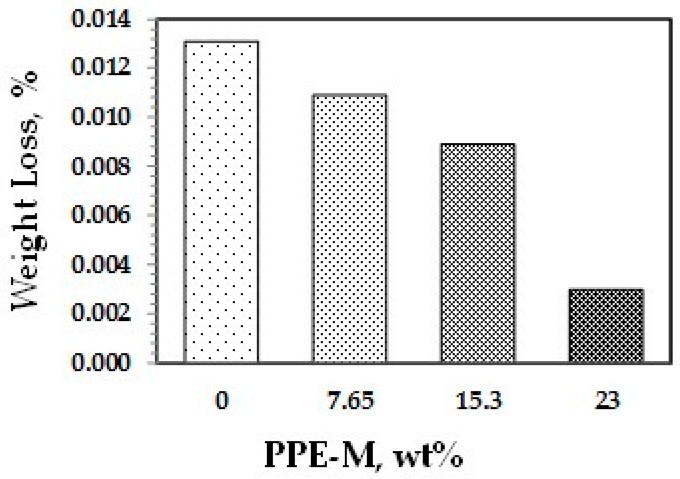
Wear resistance.

Abrasion resistance is a quantifiable property according to industry-standard Taber abrasion testing. Polyurethanes have long been recognized for their ability to resist wear in challenging applications. The effect of PPE-M on accelerated wear testing of TPUs was performed. The weight loss data using a Taber Rotary Abrader appear in Figure 36. The wear resistance of the TPUs was significantly increased with higher levels of PPE-M.

**Figure 37:**
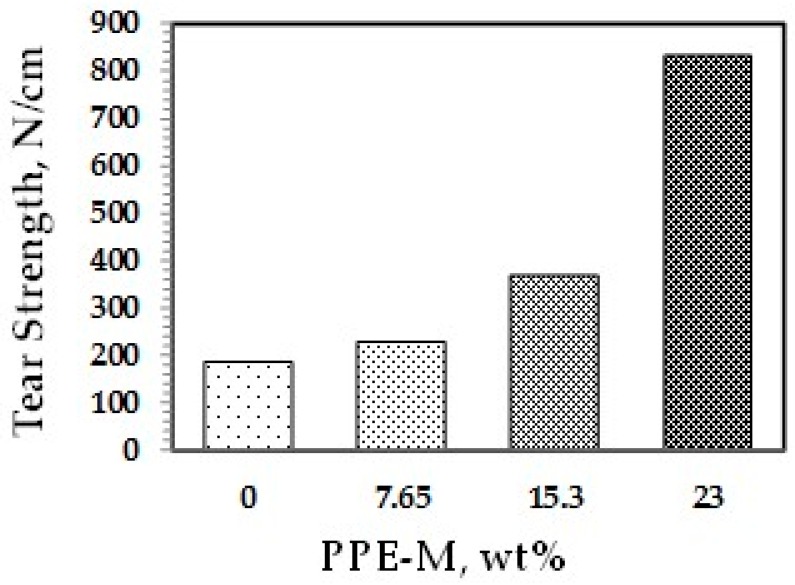
Tear resistance.

The tear strengths of the TPUs were measured according to specification ASTM D624—Die C. The test measures the resistance in a material to the formation of a tear (tear initiation) and the resistance to the expansion of a tear (tear propagation). The tear strengths were significantly greater with increased levels of PPE-M, as shown in Figure 37. PPEs engineering features of strength and toughness contribute to the enhancements in tear strength and wear resistance. 

**Figure 38:**
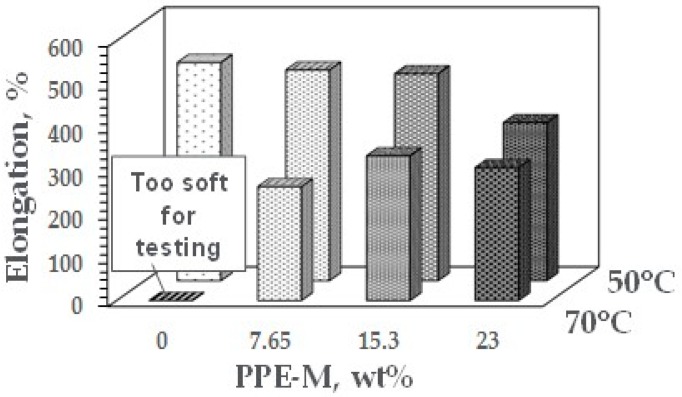
Elongation at elevated temperatures.

At ambient temperatures, all of the TPUs exhibited elongations greater than 500%. At elevated temperatures, the elongations decreased. The PPE-M based TPUs exhibited greater retention of elongation going from 50 to 75 °C. In addition, at 70 °C, the TPU with no PPE-M was too soft for testing. The results appear in Figure 38. Increased levels of PPE and its high thermal performance feature result in enhanced performance at elevated temperatures.

The aliphatic ether polyols are susceptible to oxidation and are subject to auto-oxidation. The basic mechanism for auto-oxidation of polyethers follows the scheme reported by Bolland and Gee [55]. The degradation is initiated by oxygen molecules which form peroxy radicals (P–O–O•) on the polyether [56]. In the propagation step, the peroxy radical abstracts a hydrogen from a polyether to form a hydroperoxide (P–O–O• + RH → P–O–OH + R•). The hydroperoxide will undergo thermally induced degradative reactions. The alkyl radical (R•) will react with oxygen to from a hydroperoxide (R–O–O•) and continue the auto-oxidation reactions.

PPE has a highly aromatic backbone with no aliphatic ether groups and which does not readily undergo thermo-oxidative reactions. In general, PPE based thermoplastics are used in applications exposed to elevated temperatures [1,5].

**Figure 39:**
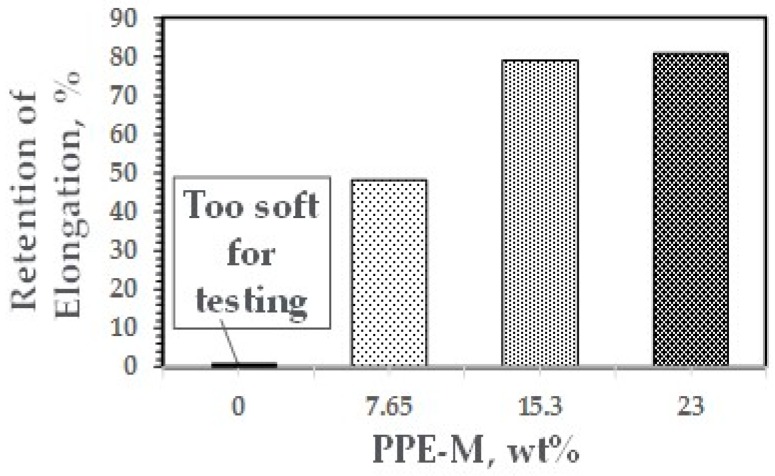
Effect of PPE-M on heat aging at 100 °C.

The enhancement of thermo-oxidative stability versus the PPE levels was measured by heat aging test parts at 100 °C for seven days and then measuring tensile properties at ambient temperature. The sample without PPE-M had deteriorated and became too soft for testing. Samples with PPE-M exhibited much greater retention of properties after heat aging, as shown in Figure 39.

**Figure 40:**
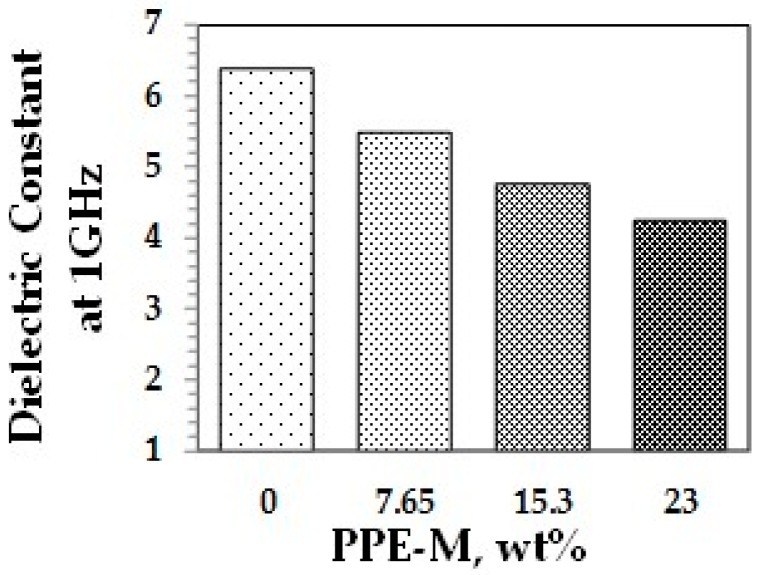
Dielectric constant.

**Figure 41:**
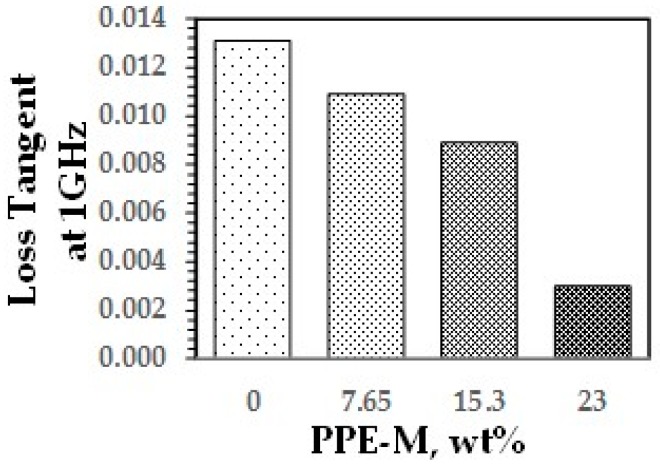
Loss tangent.

A key feature of PPE is its very low dielectric properties. The use of PPE-M in TPUs imparts greater electric insulating properties. Indeed, the dielectric constant (Dk or relative permittivity) and loss tangent (Df or dissipation factor) at 1 MHz decreased with increased PPE-M levels, as shown in Figure 40 and Figure 41.

Chemical resistance of TPUs containing PPE-M improved in both polar and non-polar solvents as well as in oil and acidic and basic solutions. The weight gain after three day of immersion in the chemicals at ambient temperatures, decreased with an increase of PPE-M levels in TPUs. Data for TPUs with 0% and 23% PPE-M are summarized in Table 5.

Thermal decomposition and combustion are an important area in some polyurethane applications. In general, polyurethanes are very combustible plastics [57]. The thermal degradation of polyether-urethanes is complex since the structure contains both polyether and urethane segments. The thermal decomposition involves the urethane linkage undergoing an O-acyl fission generally below 300 °C to generate the free isocyanate and alcohol [58]. Moreover, it has been estimated that the thermal degradation of some polyurethane products may begin as low as about 150 °C to 180 °C [59]. In addition to thermos-oxidative degradation, aliphatic polyether polyols are prone to thermal degradation where the degradation is initiated by random scission of both C‒O and C‒C bonds in the polymer chains [60].

**Figure 42:**
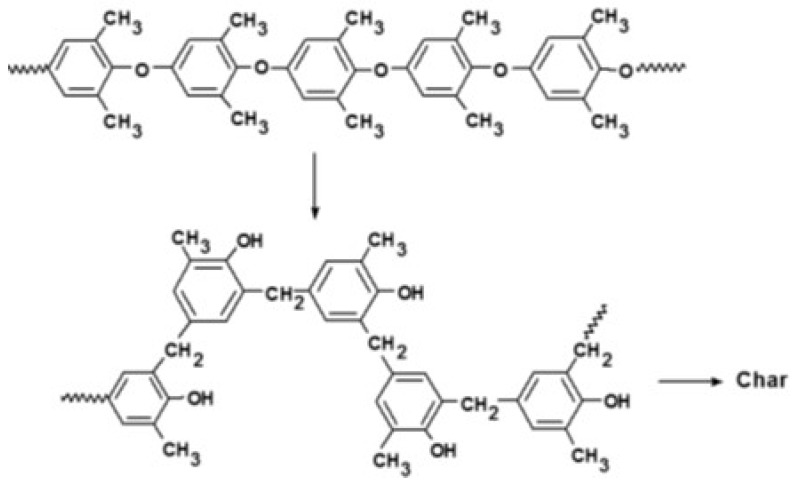
Thermal degradation mechanism of PPE.

On the other hand, PPE has an aryl ether backbone with much greater thermal stability. Studies on the thermal degradation of PPE have shown that the thermal degradation occurs in two steps [61,62,63]. The first step in the degradation occurs between 430 and 500 °C where the polymer undergoes thermal Fries-type rearrangements of the polymer chain [64]. Hence, the methyl aryl ethers undergo thermal rearrangement in the formation of benzyl phenols. The second step occurs at higher temperatures where the char-forming process results in the formation of a black, highly cross-linked residue and the evolution of phenolic products, and water. This thermal rearrangement is depicted in Figure 42.

**Figure 43:**
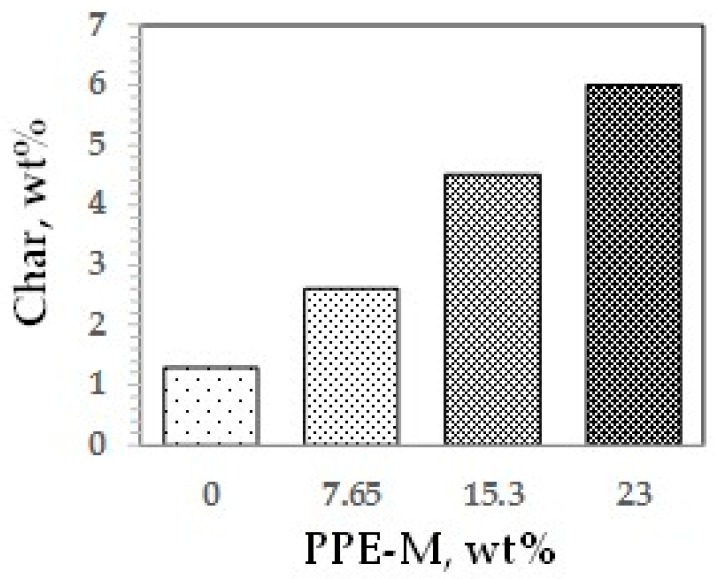
Carbonaceous residue (Char).

Polymer degradation and combustion are complex processes [65]. Two main degradation routes during pyrolysis are the formation of carbonaceous residue (char) and combustible vapors. In general, increasing the amount of char could reduce the generation of combustible gases, help to form a thermal barrier between the condensed phase and the flame, limit the heat emitted by the pyrolysis reaction, decrease the solid’s conductivity of heat, and thus reduce the flammability of materials [66]. The char of the TPUs at 800 °C was measured by thermo-gravimetric analysis in nitrogen, as shown in Figure 43. Char increased with increasing levels of PPE-M. The increased char could have implications for ease of flame retarding and meeting flammability regulations.

## 4. Conclusions

PPE macromonomers are unique hydrophobic bisphenols or diols. PPE-Ms were used to synthesize novel amphiphilic block copolymers. PPE-M was effective in broadly imparting engineering properties to PHEs and TPUs. These novel ABCs offer design engineers more flexibility in material selection. Indeed, these materials offer new options for unique combinations of performance characteristics, such as high impact strength with excellent heat resistance, lower moisture absorption, and increased mechanical and thermal properties. 

In addition to the hydrophilicity, PPE-M exhibits PPE-philicity and polystyrene-philicity. These features in combination with the polyester-philic and hydrophilicity of PHE and PU segments demonstrated utility in compatibilization of alloys and improving the resin–filler interface adhesion in composites.

A fruitful area for future ABC activity is in polymer blends and alloys. In general, blends and alloys of thermoplastics have an annual growth rate of 8–10% and constitute greater than 50% of the sales of plastics. Blends and alloys can be economically more attractive than to prepare copolymers or develop a totally new polymer. New products can be developed more rapidly by combining available polymers to produce desirable and novel [27,28]. The available degrees of freedom provide almost infinite possibilities and make the opportunity challenging. 

Properties of alloys are dependent on the nature of the polymers, interfacial attraction between the phases and the morphology. Properties can be additive (linear behavior) and based on the linear contribution from each polymer fraction. In addition, properties can exhibit a synergistic combination of properties (positive nonlinear behavior) where the properties are better than those predicted by linear behavior [25]. Clearly, interfacial adhesion is key in optimizing synergistic combination of properties [5].

Of particular interest are alloys of PPE. The PPE family of resins has become one of the most successful and best-known polymer blends and alloys. In general the PPE adds strength, lower moisture absorption, lower dielectric properties, increased dimensional stability, increased toughness, and increased thermal properties. These blends include both miscible and immiscible blends. The immiscible blends require improvements in interfacial adhesion. These PPE-philic ABCs could expand PPE alloys into new areas. For example, the PPE-philic and polyester-philic nature of PPE-PHE ABCs could enhance the performance of PPE alloys with other ester containing polymers which include recycled bottle grade of poly(ethylene terephthalate), polylactic acid, and polyethylene vinyl acetate. 

Another interesting area would be to explore the effect of ABCs on the resin–fiber adhesion in thermoset and other thermoplastic composites. For example, syndiotactic polystyrene is a semi-crystalline engineering thermoplastic and their GF grades suffered from poor mechanical properties—lower strength and stiffness, and weld line strength—relative to other engineering thermoplastics. These materials are handicapped by the lack of interfacial attraction between the glass fiber and the nonpolar, nonfunctional SPS. Thus, it would be beneficial to translate the performance enhancing capabilities of ABCs in GF–PS into SPS and other resin systems.

The data in this review showed that over the range studied, higher molecular weight PPE segments gave better properties. In blends and composites, the data suggest that higher MW segments had increased interaction between the PS and PPE phases. It would be educational to further explore the effect of molecular weight to define the optimum composition. Indeed, the PPE-PHE ABCs were homogeneous with a single Tg. Understanding where the onset of phase separation occurs, the effect of molecular weight on phase separation, and the effect on performance should be explored. 

## Figures and Tables

**Table 1:** Comparison of PPE and PPE-M.

Property	PPE	PPE-M
Mn, g/mol	15,000	1670
HEW, g/equiv.	~15,000	800
Tg, °C	215	150
Solubility at 25 °C		
2-Butanone	<1%	>50%
Toluene	~20%	>50%
Functionality	~1	~2

**Table 2:** Composition of PBT/PPE/SEBS blends.

	PBT, wt %	PPE, wt %	SEBS, wt %	PHE, wt %	ABC-36, wt %
**Blend**	40	48	12	-	-
**Blend with PHE**	40	40	10	10	-
**Blend with ABC-36**	40	40	10	-	10

**Table 3:** Composition of GF PS/PPE.

Designation	PS, wt %	GF, wt %	PHE, wt %	ABC-36, wt %
**PS/GF**	80	20	0	0
**PS/GF/PHE**	75	20	5	0
**PS/GF/ABC-36**	75	20	0	5

**Table 4:** Formulations for PPE-M/EO-PO based TPUs.

PPE-M, wt %	EO-PO, wt %	4,4′-MDI, wt %	BD, wt %	DBTDL, phr
**0**	76.70	19.99	3.31	0.0089
**7.65**	68.89	19.95	3.51	0.0038
**15.29**	61.17	20.02	3.52	0.0013
**23.00**	53.67	20.34	2.99	0.0027

**Table 5:** Weight increase (%) after immersion in chemicals for three days.

Chemical	PPE-M Content, wt %	
	0	23
**Toluene**	290	150
**Oil**	0.34	0.18
**Hydrochloric acid, pH 1**	84	19
**Sodium hydroxide, pH 13**	70	29

## Disclaimer

The materials, products and services of Saudi Basic Industries Corporation (SABIC) or its subsidiaries or affiliates (“Seller”) are sold subject to Seller’s standard conditions of sale, which are available upon request. Information and recommendations contained in this document are given in good faith. However, Seller makes no express or implied representation, warranty or guarantee: (i) that any results described in this document will be obtained under end-use conditions; or (ii) as to the effectiveness or safety of any design or application incorporating Seller’s materials, products, services or recommendations. Unless otherwise provided in Seller’s standard conditions of sale, Seller shall not be responsible for any loss resulting from any use of its materials, products, services or recommendations described in this document. Each user is responsible for making its own determination as to the suitability of Seller’s materials, products, services or recommendations for the user’s particular use through appropriate end-use and other testing and analysis. Nothing in any document or oral statement shall be deemed to alter or waive any provision of Seller’s Standard Conditions of Sale or this Disclaimer, unless it is specifically agreed to in a writing signed by Seller. Statements by Seller concerning a possible use of any material, product, service or design do not, are not intended to, and should not be construed to grant any license under any patent or other intellectual property right of Seller or as a recommendation for the use of any material, product, service or design in a manner that infringes any patent or other intellectual property right. SABIC and brands marked with ™ are trademarks of SABIC or its subsidiaries or affiliates, unless otherwise noted. Any brands, products or services of other companies referenced in this document are the trademarks, service marks and/or trade names of their respective holders.
